# Effect of an anti-adhesion agent on vision-based assessment of cervical adhesions after thyroid surgery: randomized, placebo-controlled trial

**DOI:** 10.1038/s41598-021-97919-8

**Published:** 2021-10-07

**Authors:** Hyeong Won Yu, Dongheon Lee, Keunchul Lee, Su-jin Kim, Young Jun Chai, Hee Chan Kim, June Young Choi, Kyu Eun Lee

**Affiliations:** 1 Department of Surgery, Seoul National University Bundang Hospital, Seongnam-si, Korea; 2 Department of Biomedical Engineering, Chungnam National University College of Medicine and Hospital, Daejeon, Korea; 3 Department of Surgery, Seoul National University Hospital and College of Medicine, Seoul, Korea; 4 Department of Surgery, Seoul National University Boramae Medical Center, Seoul, Korea; 5 Department of Biomedical Engineering and Institute of Medical & Biological Engineering, Medical Research Center, Seoul National University College of Medicine and Hospital, Seoul, Korea

**Keywords:** Computational biology and bioinformatics, Endocrinology, Medical research

## Abstract

Many patients experience cervical adhesions after thyroid surgery. To date, however, no studies have objectively measured the effects of anti-adhesion agents on cervical adhesion symptoms. This study evaluated the effects of an anti-adhesion agent on cervical adhesions after thyroid surgery, as determined using a system that measures the extent of marker movement objectively. One hundred patients were randomized in a 1:1 ratio to undergo thyroid surgery with or without the anti-adhesion agent Collabarrier. Using specially manufactured recording equipment, the position of the marker on neck skin was measured before surgery, and 2 weeks, 3 months, and 9 months after surgery. Relative change in marker distance, calculated by subtracting the marker position before surgery from the marker positions 2 weeks, 3 months, and 9 months after surgery, differed significantly in the groups of patients who underwent thyroid surgery with and without the anti-adhesion agent (P < 0.05). A novel measuring system can objectively evaluate the effectiveness of a thyroid anti-adhesion agent. The anti-adhesion agent used significantly reduced adhesions compared with the control group. The trial is registered at www.cris.nih.go.kr (KCT0005745; date of registration, 08/01/2021).

## Introduction

The thyroid gland is an endocrine organ located at the lower part of the neck that secretes thyroid hormones^1^. Thyroid surgery is the treatment of choice in most patients with thyroid cancer and in some patients with benign thyroid nodules^2^. Traditional thyroidectomy is performed by making an incision of about 5–7 cm in the lower skin of the neck^3^. Cervical adhesions, consisting of adhesions between the subcutaneous fascia and tissues around the airways, may occur after thyroidectomy^4^. These cervical adhesions may cause symptoms such as swallowing impairment, foreign body sensation, dysphagia, a pulling sense around the neck, and difficulties in vocalization. These adhesions may also cause abnormal skin wrinkling, reducing patient quality of life.

Various biomaterials have been shown to prevent tissue adhesions^5^, although the effects of anti-adhesive agents on cervical adhesions after thyroid surgery vary widely. For example, one study found that sodium hyaluronate carboxymethyl cellulose membranes did not reduce subjective or objective measures of postoperative adhesions in 80 patients who underwent thyroid surgery^6^, whereas another study found that acellular dermal matrix improved swallowing impairment after surgery in 40 patients^7^. More than 50% of patients who undergo thyroid surgery experience non-specific dysphasia, with about 15% experiencing these symptoms for up to 5 years^8,9^. New treatments are therefore needed to reduce cervical adhesions after thyroid surgery.

Although many studies have evaluated voice changes and hypoparathyroidism after thyroidectomy, few to date have evaluated the extent of cervical adhesions and their symptoms, or the ability of treatments to reduce adhesions. In addition, most studies have evaluated subjective symptoms because it is difficult to objectively evaluate symptoms of thyroid adhesions. Subjective surveys have disadvantages because the results may reflect the evaluator’s intentions and may depend on the integrity of the participants. These possible limitations emphasize the need to objectively evaluate cervical adhesions and their symptoms occurring after thyroid surgery.

The present study describes a system designed to objectively measure the effectiveness of the anti-adhesion agents on thyroid adhesions. This system uses video recordings to measure the distance moved by a marker on each subject’s skin^10,11^. This system, which involved taking cervical videos of patients before and after thyroidectomy, enabled measurements of the distance moved by neck wrinkles to be determined, as well as a comparison of the distances in patients randomized to undergo thyroidectomy with and without an anti-adhesion agent.

## Results

### Demographic

The basic demographic and clinical characteristics of the patient population are shown in Table 1. No important impairments or unintended effects were detected in either group.

**Table 1:** Demographic and clinical characteristics of patients who underwent thyroidectomy with and without an anti-adhesion agent.

	With anti-adhesion agent (n = 44)	Without anti-adhesion agent (n = 44)	P-value
Sex (M, %)	15 (34.1%)	17 (38.6%)	0.5
Age, year (mean ± SD)	46.66 ± 9.12	49.93 ± 9.87	0.06
**Thyroid nodule**			0.5
Malignant	39	41	
Benign	5	3	
**Surgical site**			0.5
Total thyroidectomy	14	9	
Right thyroid lobectomy	12	26	
Left thyroid lobectomy	18	9	
Size of tumor, cm (mean ± SD)	1.48 ± 1.11	1.48 ± 1.15	0.49

*SD* standard deviation.

### Effect of anti-adhesion agent on image marker distance

**Figure 1:**
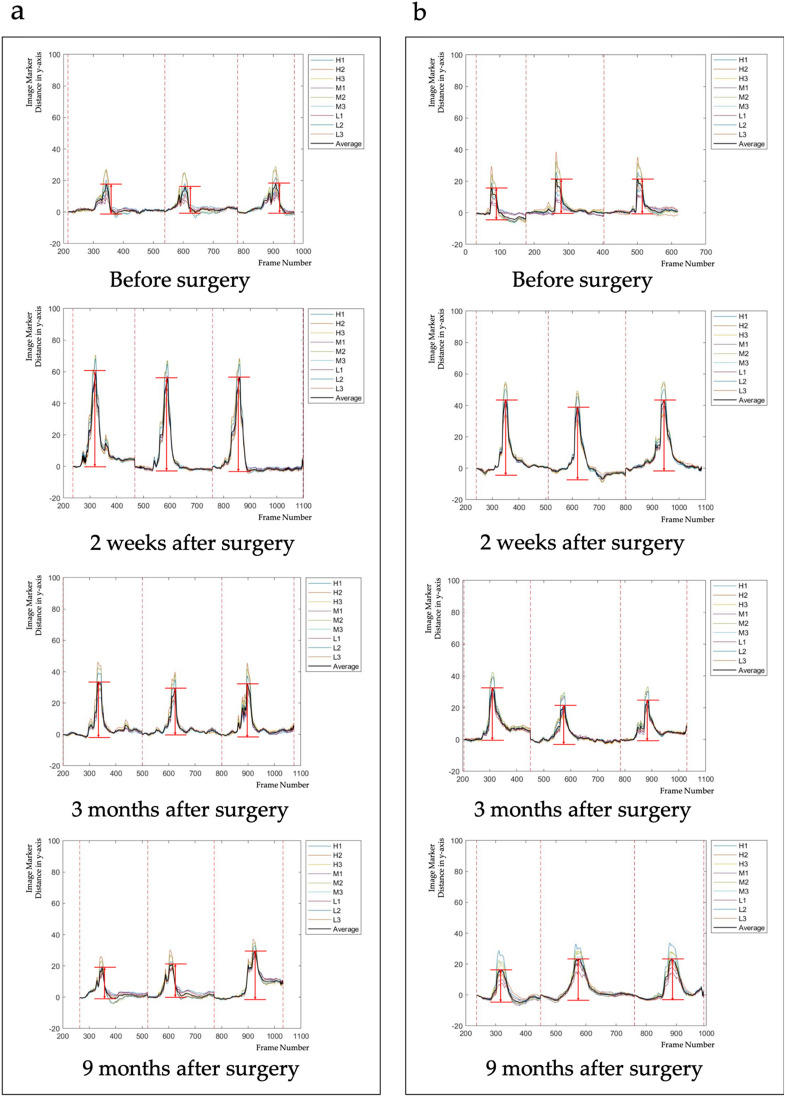
Image marker distances moved before surgery, and 2 weeks, 3 months, and 9 months after surgery in patients who underwent thyroidectomy (**a**) with and (**b**) without an anti-adhesion agent.

A comparison showed significant differences in marker distances at 2 weeks, 3 months, and 9 months between the group of patients who underwent thyroidectomy with the anti-adhesion agent and the group who underwent thyroidectomy without the anti-adhesion agent (P < 0.05 each; Fig. 1). Table 2 shows the distances the marker migrated in patients.

**Table 2:** Image marker distance migrated in patients who underwent thyroidectomy with and without an anti-adhesion agent.

	With anti-adhesion agent (n = 44)	Without anti-adhesion agent (n = 44)	P-value
**Actual distances**			
Before surgery	3.92 ± 1.19 mm	4.1 ± 1.38 mm	0.512
2 weeks after surgery	6.87 ± 2.07 mm	6.14 ± 1.84 mm	0.087
3 months after surgery	5.71 ± 1.96 mm	5.2 ± 1.65 mm	0.189
9 months after surgery	5.41 ± 2.04 mm	4.69 ± 1.72 mm	0.084
**Distances after subtracting preoperative distances**			
2 weeks after surgery	2.96 ± 2.1 mm	2.04 ± 1.85 mm	0.035
3 months after surgery	1.8 ± 1.52 mm	1.1 ± 1.55 mm	0.037
9 months after surgery	1.45 ± 1.66 mm	0.58 ± 1.5 mm	0.013

### Effect of anti-adhesion agent on marker distance as a function of sex, age, and height

Marker distance in the two groups differed significantly 3 months and 9 months after surgery in men (P < 0.05); 3 months after surgery in patients aged 50–69 (P < 0.05); and 3 months after surgery in patients > 160 cm in height (P = 0.05). Table 3 shows the effects of sex, age, and height on image marker distance in patients.

**Table 3:** Effects of sex, age, and height on the image marker distance in patients who underwent thyroidectomy with and without an anti-adhesion agent.

Sex	Male			Female		
	AA+ (n = 12)	AA− (n = 20)	P-value	AA+ (n = 29)	AA− (n = 27)	P-value
Before surgery	4.5 ± 1.12 mm	4.83 ± 1.34 mm	0.248	3.61 ± 1.11 mm	3.6 ± 1.15 mm	0.484
2 wk after surgery–Before surgery	4.5 ± 1.12 mm	4.83 ± 1.34 mm	0.11	3.62 ± 1.11 mm	3.602 ± 1.148 mm	0.072
3 mo after surgery–Before surgery	2.14 ± 1.03 mm	1.28 ± 2 mm	0.115	3.51 ± 2.22 mm	2.471 ± 1.558 mm	0.192
9 mo after surgery–Before surgery	2.26 ± 1.8 mm	0.7 ± 1.55 mm	0.007	1.74 ± 1.36 mm	1.321 ± 1.443 mm	0.116

Age, years	20–49			50–69		
	AA+ (n = 27)	AA− (n = 21)	P-value	AA+ (n = 17)	AA− (n = 23)	P-value
Before surgery	4.03 ± 1.17 mm	3.9 ± 1.15 mm	0.434	3.74 ± 1.21	4.28 ± 1.54 mm	0.149
2 wk after surgery–Before surgery	2.99 ± 2.2 mm	2.23 ± 2.05 mm	0.324	2.91 ± 1.94	1.87 ± 1.62 mm	0.059
3 mo after surgery–Before surgery	1.79 ± 1.627 mm	1.35 ± 1.48 mm	0.362	1.81 ± 1.34	0.86 ± 1.58 mm	0.033
9 mo after surgery–Before surgery	5.08 ± 18.672 mm	5.37 ± 21.21 mm	0.124	1.49 ± 1.9	0.53 ± 1.51 mm	0.063

Height	< 160 cm			> 160 cm		
	AA+ (n = 24)	AA− (n = 20)	P-value	AA+ (n = 20)	AA− (n = 24)	P-value
Before surgery	3.5 ± 0.95 mm	3.74 ± 1.19 mm	0.262	4.42 ± 1.26 mm	4.41 ± 1.46 mm	0.477
2 wk after surgery–Before surgery	3.17 ± 2.21 mm	2.09 ± 1.64 mm	0.142	2.7 ± 1.94 mm	2 ± 2 mm	0.158
3 mo after surgery–Before surgery	1.5 ± 1.06 mm	1.09 ± 1.48 mm	0.201	2.15 ± 1.88 mm	1.1 ± 1.6 mm	0.05
9 mo after surgery–Before surgery	5.33 ± 19.8 mm	0.72 ± 1.26 mm	0.158	1.72 ± 1.67 mm	4.6 ± 19.96 mm	0.056

*AA+* with anti-adhesion agent, *AA–* without anti-adhesion agent, *wk* weeks, *mo* months.

### Questionnaire results

There were no significant between-group differences in all four patient-determined items on the questionnaire, including the severity of symptoms; swallowing saliva, water, and solid objects; and the extent that wrinkles on the neck felt unnatural. In addition, there were no significant between-group differences in the three surgeon-determined items, including the extent of wrinkles at rest, the extent of wrinkles in extern, and the severity of inflammatory responses (Supplementary Table S1, Supplementary Table S2).

## Discussion

Conventional open thyroidectomy requires incisions on the lower part of the neck to remove the thyroid gland^12^. Postoperative adhesions may occur between the subcutaneous layer and larynx, or between the strap muscles and larynx^13^. About 50% of patients experience swallowing difficulty after thyroid surgery, with these difficulties lasting for > 2 years in about 15% of these patients^8,9^. Patients with severe symptoms of adhesion may experience other symptoms, including a foreign body sensation, pulling, and difficulty drinking or eating. The effectiveness of the anti-adhesion agents in reducing cervical adhesions has been found to vary among studies^6,7^. Many of these studies, however, assessed symptoms of adhesion subjectively, indicating the need for an objective measure that can assess the effects of the anti-adhesion agents.

The present study describes a method to objectively evaluate the effectiveness of an anti-adhesion agent, by measuring the movement of neck skin during swallowing using manufactured recording equipment and a vision-based image marker recognition method^14,15^. In this method, nine stickers were placed on the bottom of each patient’s neck before the operation, and 2 weeks, 3 months, and 9 months after the operation. As instructed by a recorded voice, each subject swallowed water three times, with video recordings made from the front and both sides using specially designed equipment placed at a certain distance from the patient’s neck^16^. The frontally recorded video of each patient was subjected to image processing to determine the coordinates of the image markers, which were subsequently converted from pixels to mm^17,18^. The moving distance of the marker was measured when the patient swallowed water. To confirm that any swallowing difficulty was due to the accessory muscle of the neck, recordings were also made on both sides. Because none of these patients used the accessory muscle, the side-recording videos were not analyzed.

Based on the principle that the muscles of the neck move naturally up and down when swallowing water, we assumed that the movement of the image marker along the y-axis would differ in the groups treated and not treated with the anti-adhesion agent. Observation confirmed that the movement of the image marker along the x-axis was not large. Because inter-individual differences depend on the degree of swallowing, the effect of inter-individual differences would be minimized by analyzing the differences in marker distance, i.e., by subtracting the image marker distance measured before surgery from the image marker distance measured after surgery. This analysis showed that treatment with the anti-adhesive agent reduced the number of adhesions compared with the control group. The movement of the marker in both groups was greater after than before surgery, but subsequently tended to decrease over time. Because subcutaneous fat is exfoliated to create a flap, it can move more freely than before. Patients administered the anti-adhesion agent had few or no adhesions, resulting in a significant increase in marker movement after surgery. Thereafter, the distance moved decreases over time, although the image marker still moves well. In the control group, the distance moved by the image marker slightly increased after surgery, returning to a preoperative level after 9 months.

Subgroup analyses showed that the between-group differences in marker distances were influenced by sex, age, and height. Marker distances differed significantly in men, but not in women, after 3 and 9 months; after 3 months in patients aged 50–69 years; and after 3 months in subjects > 160 cm in height. However, none of these factors was specifically associated with marker distances, suggesting that the use of an anti-adhesion agent remained important. Because the numbers of patients in individual subgroups were small, statistical comparisons were not possible. There is therefore a need for further studies, including larger numbers of patients, to determine the effects of sex, age, and height on thyroid adhesions.

In addition to the measurement of objective markers, this study also surveyed subjective outcomes among patients and surgeons. Statistical analyses confirmed that there were no differences between the two groups.

Although side-recording videos of patients can be used to analyze image markers, it is difficult to perform. In some cases, a marker located at the edge may not be visible on the screen or may not be recognized due to a sudden change in illumination during swallowing. Therefore, this study utilized the average values of the recognized marker coordinates, excluding the coordinates of unrecognized markers. In addition, in other cases, the relative positions of the top and bottom of some markers may have changed momentarily during swallowing. Thus, it was necessary to determine the marker distance obtained through the image processing. In the future, we expect to be able to fully automate the acquisition of image marker coordinates using robust machine learning techniques, allowing us to control for the effects of lighting and other characteristics^19,20^. The vision-based assessment of cervical adhesions described in this study is preliminary and will need to be applied to more subjects in the future to confirm its validity and statistical significance.

In conclusion, this study describes a novel method that can objectively evaluate adhesions after thyroid surgery by measuring the distances moved by a marker on cervical skin. This system verified the effectiveness of the anti-adhesion agent used in thyroid surgery, as it significantly reduced adhesions compared with a control group.

## Methods

### Patient

All patients provided written informed consent, and the study protocol was approved by the Research Ethics Committee of Seoul National University Bundang Hospital (E-1704-390-001, August 18th 2017). All patients provided written informed consent for publication. Trial registration was performed on Korean Clinical Trials Registry (KCT0005745, Date of registration: 08/01/2021, www.cris.nih.go.kr). All experiments were performed in accordance with relevant guidelines and regulations. The study was performed from August 18, 2017 to August 1, 2020. Patients aged 20–65 years undergoing thyroid lobectomy and total thyroidectomy for malignant or benign thyroid tumors were randomized to undergo surgery with and without an anti-adhesion agent. Patients were excluded if they had undergone previous cervical surgery; had other diseases of the esophagus or airways; had keloid wounds on the neck; had previously undergone radiation treatment of the neck; or were planning to undergo modified radical neck dissection.

### Study protocol

**Figure 2:**
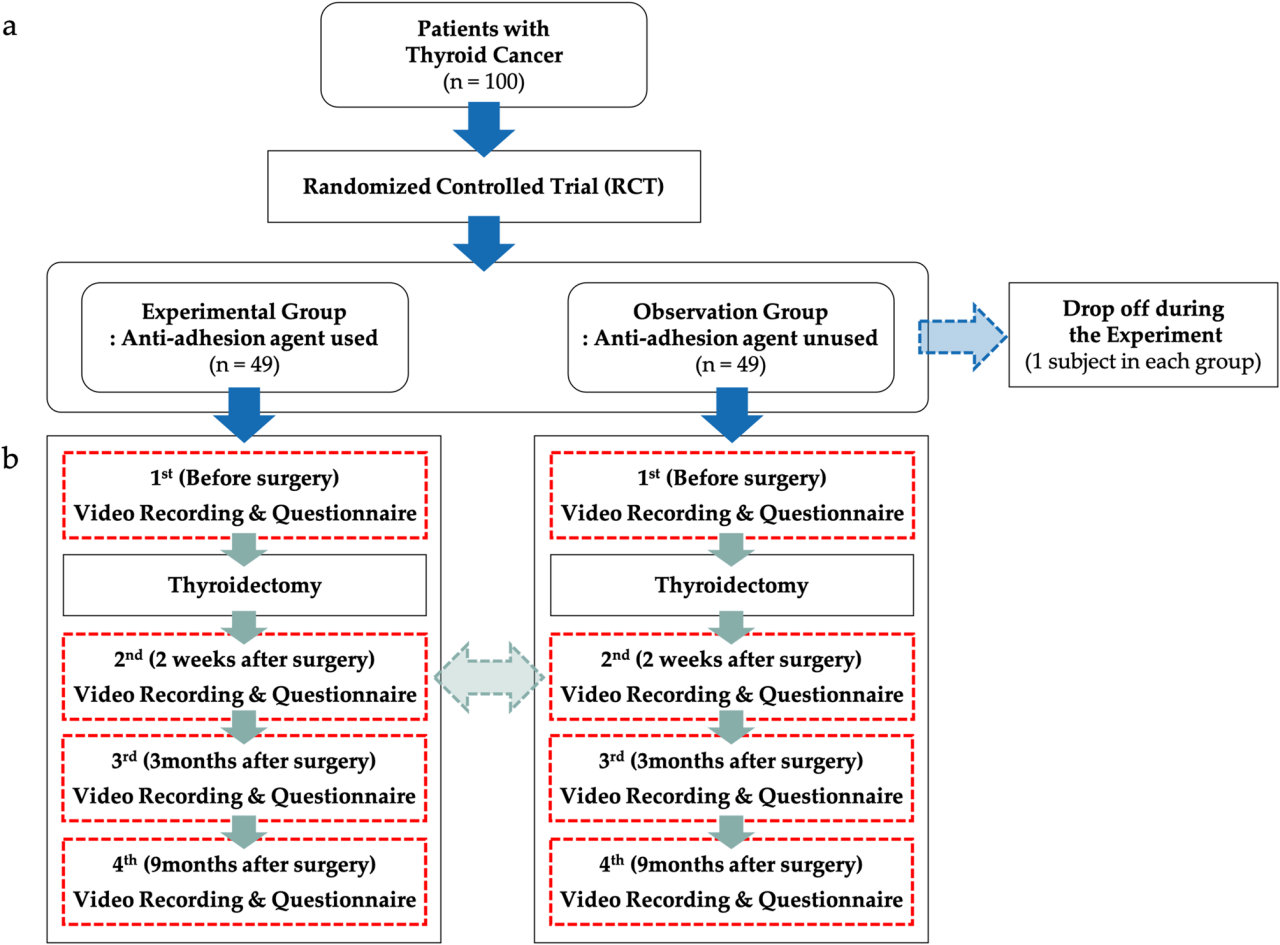
Study protocol. (**a**) Flow chart of the randomized controlled trial. (**b**) Anti-adhesion agent experiment (video recording and questionnaire) in each group.

Patients were randomized during the final steps of thyroidectomy, with the patient unaware of the assigned group. Patients were randomized and data were collected and evaluated by independent surgeons not participating in the surgery. Patients who wished to withdraw from the study were withdrawn immediately. Figure 2 shows the study flow diagram.

A total of 100 patients were randomized 1:1 to undergo thyroidectomy with and without Collabarrier, a transparent and highly viscous gel-type anti-adhesive agent (DalimTissen Co., Ltd., Seoul, South Korea) based on type I atelocollagen that is used in the suture process at the end of surgery. A doctor who did not participate in the surgery randomly assigned the patients using the random number generation method of the R studio program. Each patient randomized to Collabarrier received a total of two agents (6 cc), 3 cc divided between the trachea and strap muscles, and 3 cc divided between the strap muscles and skin flaps. Anti-adhesion agents were omitted from the suture process in patients randomized to the control group. Each included patient was video-recorded four times under the same conditions: before surgery, and 2 weeks, 3 months, and 9 months after surgery. Surveys were administered to all patients at the same time.

**Figure 3:**
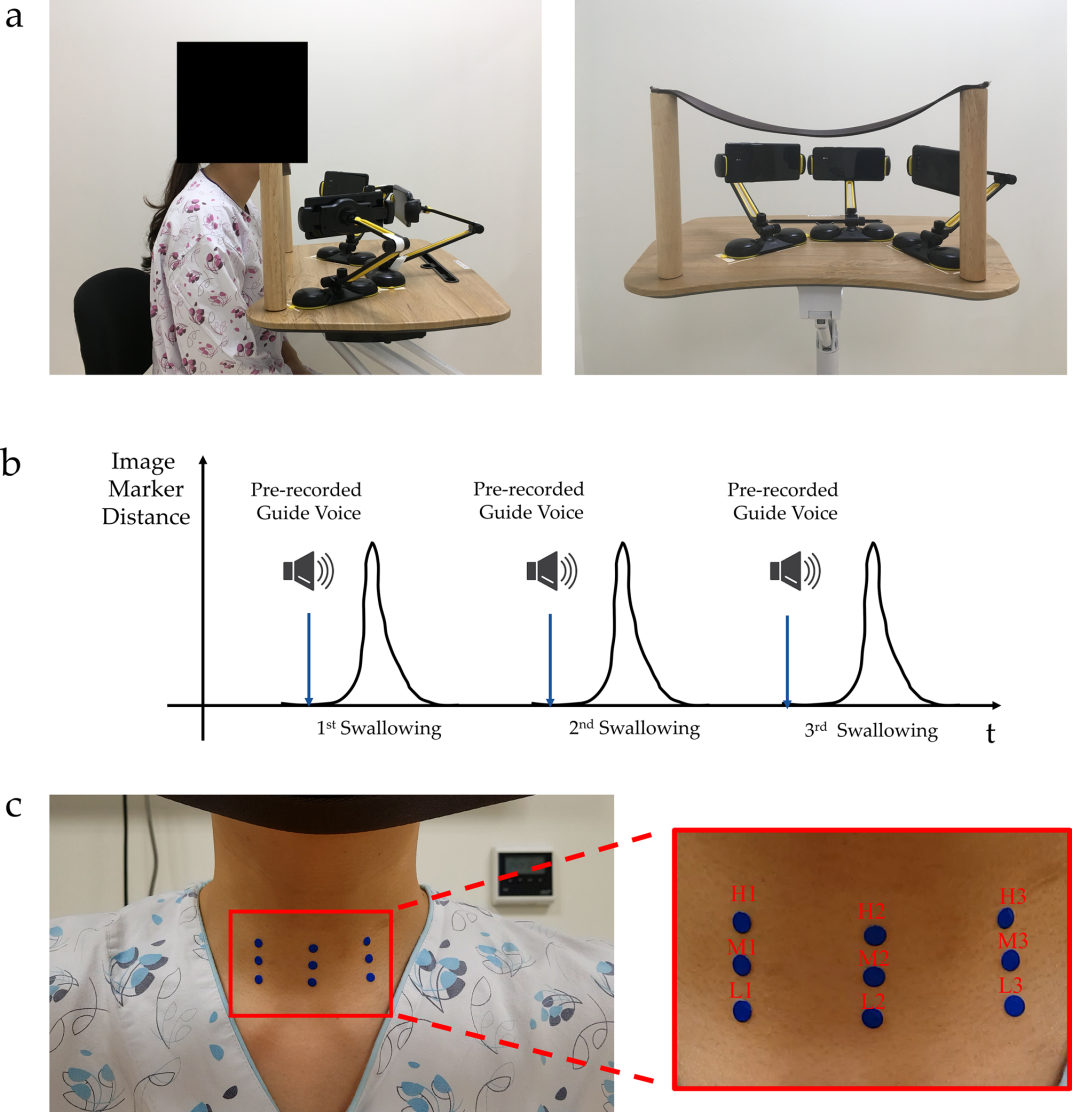
Video recording of a patient. (**a**) Imaging equipment. (**b**) Guidance by the pre-recorded voice. (**c**) Design of the image markers in the recorded video.

The video measurement system included a special video recording table that allowed patients to sit on a chair and record the surgical site^16^. The height of the table could be adjusted according to the patient’s sitting height. A ninth generation Samsung Galaxy Note (Samsung, Seoul, South Korea) was fixed at a distance of about 25 cm in front of and on both sides of the patient’s neck (Fig. 3a). Patients were asked to swallow water three times by a pre-recorded guide voice (Fig. 3b). Three swallowings were recorded simultaneously for an average of 36 s with 30 fps. Because the recordings followed the voice guide, the recording speed and intervals were constant.

### Description of the image markers

Because there are no special identification devices on the neck, nine stickers were attached as imaging markers to record neck movement. Three stickers, designated H1, H2, and H3, were each attached 1 cm above the cervical wound; three, designated M1, M2, and M3, were each attached along the cervical wound; and three, designated L1, L2, and L3, were each attached to 1 cm below the cervical wound (Fig. 3c).

### Image processing for determining image marker distance

**Figure 4:**
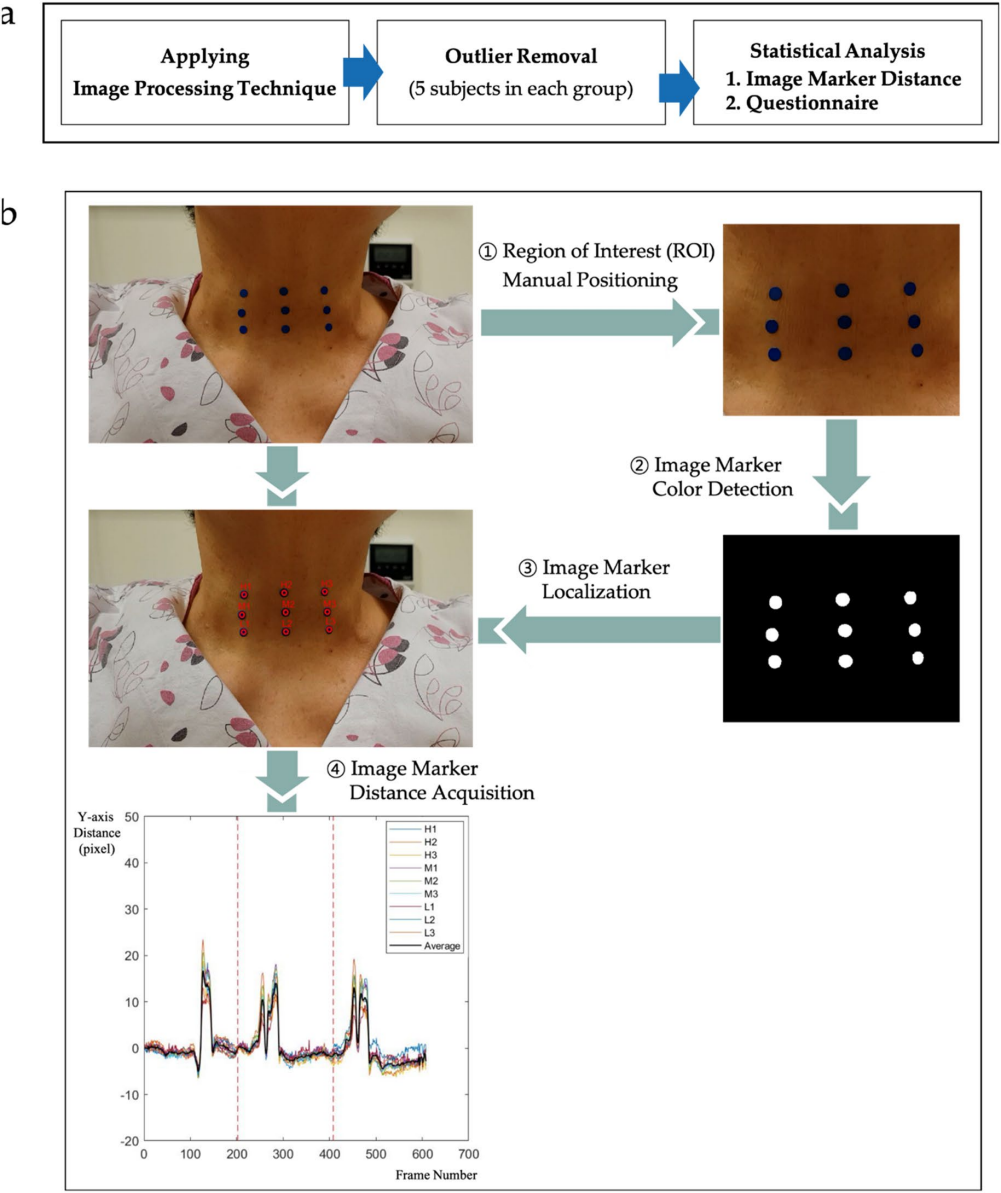
Entire process of determining and analyzing image marker movement. (**a**) Analysis of image marker movement. (**b**) Image processing technique for determining image marker movement.

The motion of each image marker in the recorded videos was determined by recognizing the marker using an image processing technique. First, the markers displayed on the first frame were manually designated the region of interest (ROI). Second, RGB color space was converted to HSV color space^14,15^, allowing recognition of the color of the markers used in this experiment. Four color markers (blue, orange, green, and yellow) were recognized, and each of the nine regions was segmented. Third, these nine marker regions were localized in order from top to bottom as high, middle, and low, and from left to right as 1, 2, and 3. Each subject was asked to swallow three times, during which the images were processed by MATLAB software (MATLAB R2017a; MathWorks Inc., Natick, MA, USA) to obtain the coordinates of the markers in the y-axis. Figure 4 shows the entire process of image marker distance acquisition and analysis.

### Evaluation of image marker distance

The marker coordinates on the y-axis were converted to mm, as a subtle difference in distance between the camera and the subject may affect the distance that the marker actually moves. To convert from pixels to mm, the diameter of the marker was pre-measured in mm during the first frame and matched to the diameter of the marker in pixels, which appeared on the screen^17,18^. The measured diameter of the marker was 5 mm.

The marker distance was defined as the difference between the maximum and minimum values along the y-axis during a swallow. The average motion of the nine markers was calculated, as was the median marker distance of each subject during three swallows. The median was calculated rather than the mean because the latter does not reflect the actual marker distance, with some errors occurring when markers moved more or less than usual during swallowing.

Subjects participating in the experiment were assumed to have inter-individual differences in neck muscles during swallowing. To control for errors caused by inter-individual differences, the difference in marker distance was measured before and after surgery by subjecting marker positions before surgery from marker positions 2 weeks, 3 months, and 9 months after surgery.

### Analysis of the image marker distance

The analysis of the results was conducted by a separate medical staff member who was not in charge of randomization or involved in the operation. Statistical analysis was performed to determine whether marker distance differed significantly between the 49 patients who were administered thyroid anti-adhesion agent and the 49 who were not. Five subjects in each group were outliers, showing an extreme marker distance statistically (P-value), and were excluded from the analysis. Image marker distance, calculated as the distance after surgery minus the distance before surgery, was compared between the two groups by independent t-tests. Market distances were compared between the two groups of patients sub-grouped by sex, age, and height by Mann–Whitney U-tests.

### Questionnaires

Patients were administered symptom questionnaires before surgery, and at 2 weeks, 3 months, and 9 months after surgery (Supplementary Table S1, Supplementary Table S2). These questionnaires evaluated the level of symptoms, on a scale from 0 to 10, when swallowing saliva, water, and solid foods such as rice. These questionnaires also evaluated the extent to which neck wrinkles appeared unnatural to study subjects when looking in a mirror.

Surgeons were also administered questionnaires before surgery, and 2 weeks, 3 months, and 9 months after surgery, asking them to evaluate the extent of wrinkles in the resting state, neck extension state, and inflammatory state. All responses were evaluated on a scale of 0–10. The questionnaires were evaluated by an independent surgeon, and all outcomes were compared between the two groups by t-tests.

## Supplementary Information

Supplementary Information.

Supplementary Video 1.

## Data Availability

The data that supports the findings are available upon request from the corresponding author.
