# Supplementary material for: Effect of an anti-adhesion agent on vision-based assessment of cervical adhesions after thyroid surgery: randomized, placebo-controlled trial
Source: Sci Rep. 2021 Oct 7;11:19935. doi: 10.1038/s41598-021-97919-8 (PMC8497539; doi:10.1038/s41598-021-97919-8)
Supplement: Supplementary file 1 — Supplementary Information. [file 41598_2021_97919_MOESM1_ESM.docx]

**Effect of an anti-adhesion agent on vision-based assessment of cervical adhesions after thyroid surgery: Randomized, placebo-controlled trial**

Hyeong Won Yu^1,†^, Dongheon Lee^2,†^, Keunchul Lee^1^, Su-jin Kim^3^, Young Jun Chai^4^, Hee Chan Kim^5^, June Young Choi^1^, and Kyu Eun Lee^3^

†These authors contributed equally to this work.

^1^Department of Surgery, Seoul National University Bundang Hospital, Seongnam-si, Korea

^2^Biomedical Research Institute, Seoul National University Hospital, Seoul, Korea

^3^Department of Surgery, Seoul National University Hospital and College of Medicine, Seoul, Korea

^4^Department of Surgery, Seoul National University Boramae Medical Center, Seoul, Korea

^5^Department of Biomedical Engineering, College of Medicine, and Institute of Medical & Biological Engineering, Medical Research Center, Seoul National University, Seoul, Korea

**Corresponding authors**

June Young Choi

Seoul National University Bundang Hospital

82, Gumi-ro 173 Beon-gil, Bundang-gu, Seongnam-si, Gyeonggi-do, 13620, Korea

Tel: 82-31-787-7107; Fax: 82-31-787-4078; E-mail: [juneychoi@snubh.org](mailto:juneychoi@snubh.org)

Supplementary Materials

**Video S1.** Procedure for determining image marker coordinates in the video record.

**Table S1**. Questionnaire results in the two patient groups (t-tests)

|  | Discomfort when swallowing saliva (Patient answer) | |  | Discomfort when swallowing water (Patient answer) | |  |
| --- | --- | --- | --- | --- | --- | --- |
|  | AA+ (n=44) | AA- (n=44) | p-value | AA+ (n=44) | AA- (n=44) | p-value |
| Before surgery | 0.45 ± 1.27 | 0.66 ± 1.76 | 0.538 | 0.39 ± 1 | 0.25 ± 1.09 | 0.548 |
| 2wk after surgery – Before surgery | 0.34 ± 1.89 | 0.43 ± 2.04 | 0.831 | 0.45 ± 1.8 | 0.57 ± 1.74 | 0.767 |
| 3mo after surgery – Before surgery | 0.16 ± 1.81 | 0 ± 1.71 | 0.676 | -0.09 ± 1.24 | 0.16 ± 1.68 | 0.434 |
| 9mo after surgery – Before surgery | 0.05 ± 1.71 | -0.3 ± 2.07 | 0.403 | -0.19 ± 1.17 | -0.07 ± 1.23 | 0.657 |
|  | | | | | | |
|  | Discomfort when swallowing solid objects (Patient answer) | |  | Appearance of wrinkles on the neck (Patient answer) | |  |
|  | AA+ (n=44) | AA- (n=44) | p-value | AA+ (n=44) | AA- (n=44) | p-value |
| Before surgery | 0.3 ± 0.87 | 0.32 ± 1.29 | 0.924 | 0.23 ± 1.49 | 0 ± 0 | 0.32 |
| 2wk after surgery – Before surgery | 0.14 ± 1.34 | 0.77 ± 2.35 | 0.127 | 0.52 ± 1.5 | 1.18 ± 2.01 | 0.089 |
| 3mo after surgery – Before surgery | 0 ± 1.3 | 0.11 ± 1.82 | 0.74 | 0.5 ± 1.47 | 0.7 ± 1.7 | 0.552 |
| 9mo after surgery – Before surgery | -0.07 ± 1.02 | -0.16 ± 1.41 | 0.73 | 1.49 ± 2.23 | 0.93 ± 1.76 | 0.207 |
|  | | | | | | |
|  | Appearance of wrinkles on the neck in resting state (Surgeon answer) | |  | Appearance of wrinkles in neck extension (Surgeon answer) | |  |
|  | AA+ (n=44) | AA- (n=44) | p-value | AA+ (n=44) | AA- (n=44) | p-value |
| Before surgery | 0.05 ± 0.21 | 0.02 ± 0.15 | 0.562 | 0.09 ± 0.36 | 0.02 ± 0.15 | 0.252 |
| 2wk after surgery – Before surgery | 0 ± 0.3 | 0.09 ± 0.42 | 0.25 | -0.07 ± 0.39 | 0.09 ± 0.42 | 0.072 |
| 3mo after surgery – Before surgery | -0.02 ± 0.26 | 0.09 ± 0.47 | 0.168 | -0.07 ± 0.39 | 0.07 ± 0.39 | 0.111 |
| 9mo after surgery – Before surgery | 0.19 ± 1.53 | 0.02 ± 0.34 | 0.502 | 0.14 ± 1.56 | 0.02 ± 0.34 | 0.639 |
|  | | | | | | |
|  | Inflammatory condition (Surgeon answer) | |  |  | | |
|  | AA+ (n=44) | AA- (n=44) | p-value |  |  |  |
| Before surgery | 0 | 0 | N/A |  |  |  |
| 2wk after surgery – Before surgery | 0.02 ± 0.15 | 0.05 ± 0.21 | 0.562 |  |  |  |
| 3mo after surgery – Before surgery | 0 | 0 | N/A |  |  |  |
| 9mo after surgery – Before surgery | 0 | 0 | N/A |  |  |  |

AA+, with anti-adhesion agent; AA–, without anti-adhesion agent; wk, weeks; mo, months.

**Table S2.** Description of the questionnaire

| Q1 | Discomfort when swallowing saliva (Patient answer) | | | | | | | | | | |
| --- | --- | --- | --- | --- | --- | --- | --- | --- | --- | --- | --- |
|  | 0 | 1 | 2 | 3 | 4 | 5 | 6 | 7 | 8 | 9 | 10 |
|  | No symptom ------------------------------------------------------------- Severe discomfort | | | | | | | | | | |
| Q2 | Discomfort when swallowing water (Patient answer) | | | | | | | | | | |
|  | 0 | 1 | 2 | 3 | 4 | 5 | 6 | 7 | 8 | 9 | 10 |
|  | No symptom ------------------------------------------------------------- Severe discomfort | | | | | | | | | | |
| Q3 | Discomfort when swallowing solid objects (Patient answer) | | | | | | | | | | |
|  | 0 | 1 | 2 | 3 | 4 | 5 | 6 | 7 | 8 | 9 | 10 |
|  | No symptom --------------------------------------------------------------- Severe symptom | | | | | | | | | | |
| Q4 | Appearance of wrinkles on the neck (Patient answer) | | | | | | | | | | |
|  | 0 | 1 | 2 | 3 | 4 | 5 | 6 | 7 | 8 | 9 | 10 |
|  | Natural -------------------------------------------------------------------------- Unnatural | | | | | | | | | | |
| Q5 | Appearance of wrinkles on the neck in resting state (Surgeon answer) | | | | | | | | | | |
|  | 0 | 1 | 2 | 3 | 4 | 5 | 6 | 7 | 8 | 9 | 10 |
|  | Natural -------------------------------------------------------------------------- Unnatural | | | | | | | | | | |
| Q6 | Appearance of wrinkles in neck extension (Surgeon answer) | | | | | | | | | | |
|  | 0 | 1 | 2 | 3 | 4 | 5 | 6 | 7 | 8 | 9 | 10 |
|  | Natural -------------------------------------------------------------------------- Unnatural | | | | | | | | | | |
| Q7 | Inflammatory condition (Surgeon answer) | | | | | | | | | | |
|  | 0 | 1 | 2 | 3 | 4 | 5 | 6 | 7 | 8 | 9 | 10 |
|  | No inflammation ---------------------------------------------------- Severe inflammation | | | | | | | | | | |

Q; question
